# Solute Carrier Family 26 Member 4 (SLC26A4), A Potential Therapeutic Target for Asthma

**DOI:** 10.35534/jrbtm.2024.10011

**Published:** 2024-06-25

**Authors:** Vineeta Guntupalli, Rongjun Wan, Liyuan Liu, Wenjing Gu, Shaobing Xie, Peisong Gao

**Affiliations:** 1 Division of Allergy and Clinical Immunology, Johns Hopkins University School of Medicine, Baltimore, MD 21224, USA; 2 Department of Respiratory and Critical Care Medicine, Xiangya Hospital, Central South University, Changsha 410008, China; 3 Department of Respiratory Medicine, Children's Hospital of Soochow University, Suzhou 215000, China; 4 Department of Otolaryngology Head and Neck Surgery, Xiangya Hospital of Central South University, Changsha 410008, China

**Keywords:** SLC26A4, Pendrin, Asthma, Transporter, Therapeutics

## Abstract

Asthma is a prevalent respiratory condition with multifaceted pathomechanisms, presenting challenges for therapeutic development. The SLC (Solute Carrier) gene family, encompassing diverse membrane transport proteins, plays pivotal roles in various human diseases by facilitating solute movement across biological membranes. These solutes include ions, sugars, amino acids, neurotransmitters, and drugs. Mutations in these ion channels have been associated with numerous disorders, underscoring the significance of SLC gene families in physiological processes. Among these, the SLC26A4 gene encodes pendrin, an anion exchange protein involved in transmembrane transport of chloride, iodide, and bicarbonate. Mutations in SLC26A4 are associated with Pendred syndrome. Elevated SLC26A4 expression has been linked to airway inflammation, hyperreactivity, and mucus production in asthma. Here, we review novel insights from SLC gene family members into the mechanisms of substrate transport and disease associations, with specific emphasis on SLC26A4. We explore triggers inducing SLC26A4 expression and its contributions to the pathogenesis of pulmonary diseases, particularly asthma. We summarize the inhibitors of SLC26A4 that have shown promise in the treatment of different phenotypes of diseases. While SLC26A4 inhibitors present potential treatments for asthma, further research is imperative to delineate their precise role in asthma pathogenesis and develop efficacious therapeutic strategies targeting this protein.

## 1. Introduction

The prevalence of asthma continues to pose significant challenges in therapeutic development, as its multifaceted pathomechanisms encompass intricate interactions between genetic predispositions, environmental triggers, and immune dysregulation [1,2]. It is estimated to affect at least 300 million individuals globally, 20% of these individuals had one or more asthma attacks within the past 12 months and experienced severe cases, so targeted therapeutic interventions are imperative for effective management [3]. While inhaled corticosteroids (ICSs) are used as the primary treatment for bronchial asthma, there is a significant challenge: a subset of asthma patients, approximately 5–10%, exhibit resistance or hypo-responsiveness to ICS therapy [4,5]. Furthermore, a significant drawback of molecularly targeted drugs, particularly biologics, is that they tend to be expensive, adding to the economic burden of asthma management [5,6]. Consequently, there is an urgent need to deepen our understanding of the molecular mechanisms underlying asthma pathogenesis and develop novel therapeutic agents based on these mechanisms to provide more effective and affordable treatments.

There is a growing interest in the Solute Carrier (SLC) gene family because of their pivotal roles in various diseases, including respiratory disorders like asthma and chronic obstructive pulmonary disease (COPD) [7,8]. A crucial aspect of the SLC gene family is its involvement in ion transport, particularly through a subset of SLC gene families known as ion channels. These channels regulate the movement of essential ions such as protons, sodium, potassium, and chloride across cell membranes, playing a critical role in maintaining cellular pH, membrane potential, and osmolarity. Dysregulation or mutations in these ion channels can lead to numerous disorders through genome-wide association studies, underscoring the significance of SLC gene families in physiological processes [9]. For example, SLC6A4 (the serotonin transporter) is involved in the reuptake of serotonin, and its functional polymorphisms have been associated with the incidence of schizophrenia [10]. Mutations in SLC2A2 (glucose transporter 2) are linked to Fanconi-Bickel syndrome, a rare inherited disorder characterized by the inability to properly metabolize glucose and glycogen [11]. Similarly, mutations in SLC26A3 are associated with congenital chloride diarrhea [12,13]. One prominent member of the SLC gene family, SLC26A4 (sulfate-chloride exchangers), encodes pendrin, an anion exchange protein involved in the transmembrane transport of chloride, iodide, and bicarbonate ions, and mutations in SLC26A4 have been associated with Pendred syndrome [14]. SLC26A4 is particularly relevant in the context of respiratory diseases such as asthma, where aberrant ion transport contributes to airway inflammation, hyperreactivity, and mucus hypersecretion [15–19]. Of interest, SLC26A4 has been identified as a common mediator for mucus production in bronchial asthma and COPD [16]. However, the mechanisms underlying the induction of SLC26A4 expression in response to various triggers, such as allergens, pollutants, and inflammatory mediators, remain an area of active investigation. Understanding these triggers and the downstream signaling pathways involved could provide insights into the development of targeted therapies for asthma. Inhibition of SLC26A4 has shown promise in preclinical studies for attenuating airway hyperreactivity, reducing mucin expression, and dampening lung inflammation, highlighting its potential as a therapeutic target in asthma management. However, translating these findings into clinical applications requires a deeper understanding of SLC26A4's precise role in asthma pathogenesis, as well as the development of efficacious and safe therapeutic strategies targeting this protein.

This review aims to consolidate existing literature on the SLC gene family, with a specific focus on SLC26A4, elucidating its mechanisms of substrate transport and its associations with respiratory diseases, notably asthma. By examining the current state of knowledge and identifying gaps in understanding, this review aims to stimulate further research into the role of SLC26A4 in asthma pathogenesis and the development of novel therapeutic interventions to alleviate asthma symptoms and improve patient outcomes.

## 2. SLC Gene Family and Its Role in Ion Transport

The SLC gene superfamily, one of the largest gene families in the human genome, comprises 65 families encompassing 458 transporters based on sequence similarity, function, and substrate specificity [20]. The SLC gene family is responsible for encoding membrane transport proteins that facilitate the movement of diverse substances, such as ions, amino acids, sugars, nucleotides, vitamins, metabolites, toxins, and drugs across cell membranes with transport often driven by internal or external ion or metabolite gradients [20]. These proteins play crucial roles in maintaining cellular homeostasis by regulating the transport of these various solutes into and out of cells [21].

**Figure 1.**
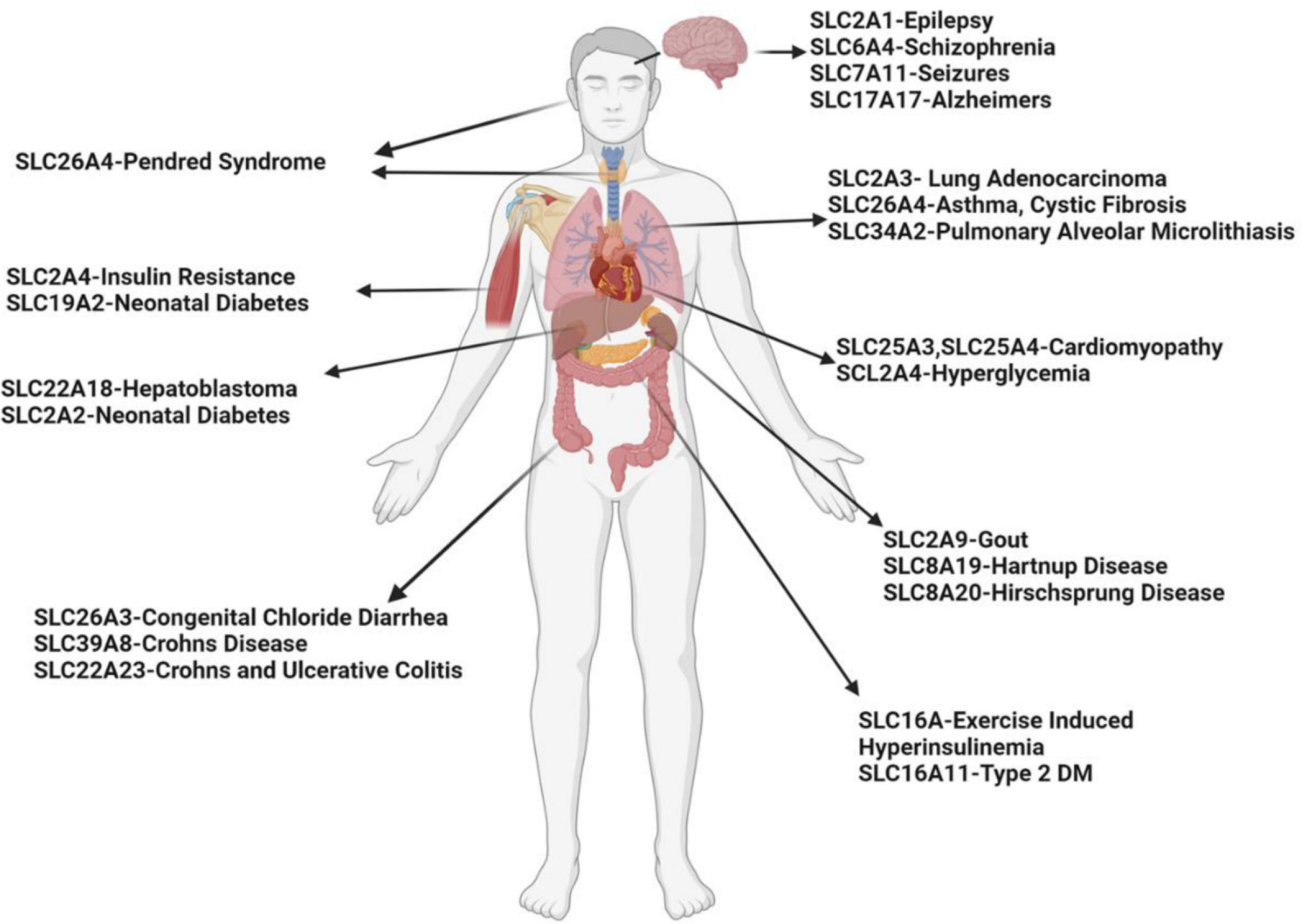
Expression of the SLC gene superfamily proteins and their associations with different diseases.

The SLC gene superfamily proteins are widely expressed throughout the body, most notably in the epithelia of major organs (e.g., liver, intestine, kidney) and organs with barrier functions (e.g., skin, brain, testes, and placenta) and are involved in a wide range of physiological processes in a variety of diseases (Figure 1) [21,22]. Different transporters are localized to the plasma membrane and to membranes that compose various subcellular organelles, ensuring the successful delivery of required substrates and thereby cellular homeostasis [23]. Many transporters are also expressed in an organ-specific manner, and facilitate the entry and elimination of endogenous and xenobiotic compounds, including nutrient uptake, ion transport, waste removal, and drug absorption and excretion. They also function as receptors for inflammatory and metabolic signaling at the lysosome and endosomal membranes [24]. Furthermore, SLC-like proteins, such as the KDEL receptor (KDELRs, ubiquitous seven-transmembrane domain proteins encoded by three mammalian genes) [25], have emerged as essential regulators of protein trafficking between endoplasmic reticulum (ER), Golgi, and plasma membrane by coupling ligand binding to pH changes in the secretory pathway and forming dynamic interactions with cytoplasmic coat protein complexes, coat protein complex I (COPI), and COPII [24]. COPI and II are essential, highly conserved pathways that traffic proteins and lipids between the endoplasmic reticulum (ER) and the Golgi [26]. Additionally, it is worth noting that functional redundancy within the SLC superfamily genes has been suggested for a number of metabolite classes [9,27]. Inhibition of one SLC often leads to expressional or a pharmacokinetic change in other SLC family members [26], and similarly, loss of certain SLCs may trigger metabolic compensations via SLCs crosstalk [28,29]. These findings indicate the presence of an integrated and tightly regulated regulatory network governing transporter functional networks.

**Figure 2.**
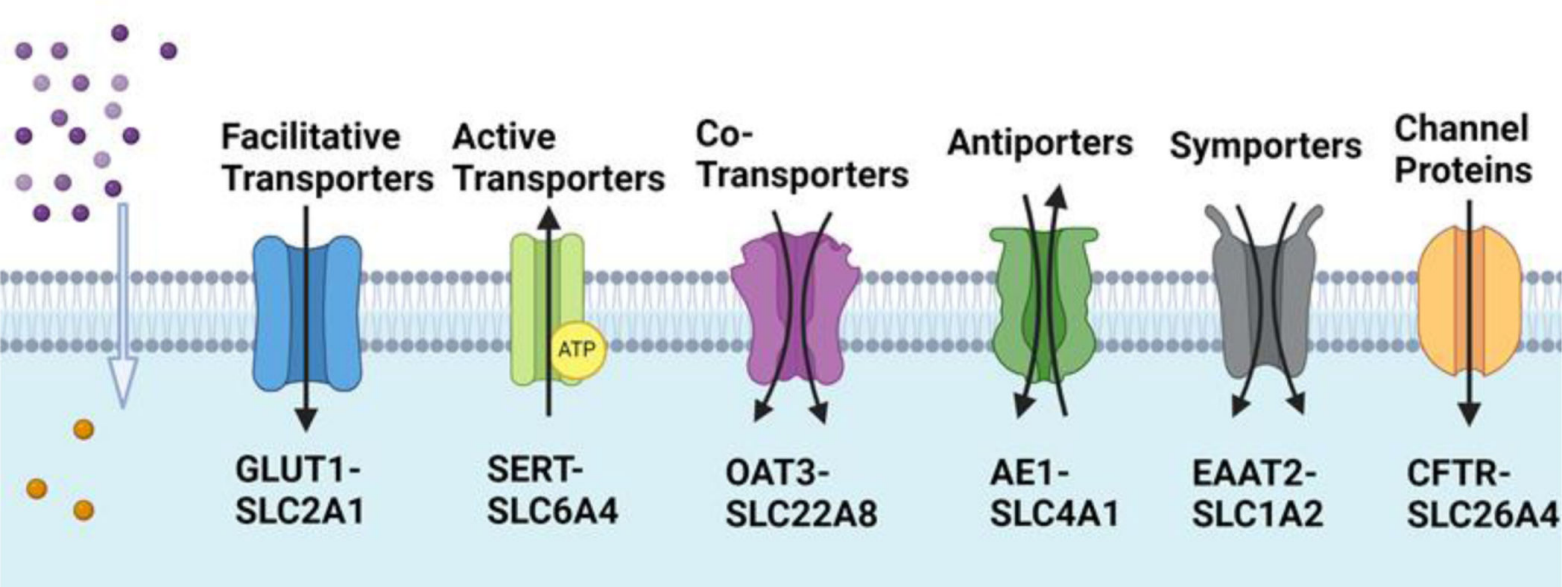
Major SLC-transporters expressed in the plasma membrane facilitate the movement of substrates across cell membranes.

The SLC gene family encompasses a wide array of transport mechanisms to facilitate the movement of substrates across cell membranes. As illustrated in Figure 2, there are several major SLC-transporters expressed in the plasma membrane, including facilitative transporters, active transporters, secondary active transporters, antiporters, symporters, co-transporters, and channel proteins [21].

For example, facilitative transporters facilitate the movement of substrates passively down along their concentration gradients without requiring energy input, such as glucose transporters and some amino acid transporters (e.g., SLC2A1/GLUT1) [30]. SLC2A1, also known as GLUT1 (Glucose Transporter 1), plays a crucial role in facilitating the transport of glucose across cell membranes [31]. While SLC2A1 is expressed in various tissues throughout the body, it is particularly abundant in tissues with high glucose utilization rates, such as the brain, red blood cells, and the blood-brain barrier endothelial cells [32]. SLC2A1 has been the subject of extensive research due to its importance in glucose metabolism and its association with various diseases. Active transporters utilize energy to transport substrates against their concentration gradient by the sodium-potassium pump (Na^+^/K^+^-ATPase) and calcium pumps (e.g., SLC6A4/SERT) [33]. SLC6A4, also known as the serotonin transporter (SERT), is a member of the SLC6 family of neurotransmitter transporters [34]. It plays a crucial role in the reuptake of serotonin from the synaptic cleft into presynaptic neurons, terminating neurotransmission and regulating serotonin signaling. Dysfunction or dysregulation of SLC6A4 can lead to alterations in serotonin levels and signaling, which have been implicated in various neuropsychiatric disorders, including depression, anxiety disorders, schizophrenia, and obsessive-compulsive disorder (OCD) [10,33,35,36]. Antiporters transport two different molecules in opposite directions (one substrate moves into the cell, another moves out) across the membrane (e.g., SLC4A1/AE1). SLC4A1, known as anion exchanger 1 (AE1) and also recognized as a chloride/bicarbonate exchanger (Cl^−^/HCO_3_^−^ exchanger), plays an essential role in maintaining the correct acid levels (pH) in the body [37]. Mutations in the gene have been associated with the development of distal renal tubular acidosis characterized by the failure to acidify the urine, resulting in nephrocalcinosis and renal failure [38]. In contrast, symporters transport two different molecules in the same direction across the membrane (e.g., SLC1A2/EAAT2) [21]. SLC1A2 is a gene that encodes excitatory amino acid transporter 2 (EAAT2), also known as glutamate transporter 1 (GLT-1). This protein is the principal transporter that clears the excitatory neurotransmitter glutamate from the extracellular space at synapses in the central nervous system [39]. Glutamate clearance is necessary for proper synaptic activation and to prevent neuronal damage from excessive activation of glutamate receptors. The dysfunction of SLC1A2 has been implicated in various neurological disorders, including epilepsy, schizophrenia, and amyotrophic lateral sclerosis (ALS) [39–41]. Intriguingly, SLC1A2 has recently been shown to play a critical role in inflammatory macrophage polarization [42]. Notably, lysosomal EAAT2 transports Glu and Asp from the lysosomes to cytoplasm to activate V-ATPase, which supports micropinocytosis and mTORC1 signaling to sustain inflammatory macrophage polarization. Co-transporters transport two or more molecules simultaneously, which can be either symporters or antiporters, depending on the direction of substrate movement (e.g., SLC22A8/OAT3) [21]. SLC22A8, also known as organic anion transporter 3 (OAT3), is responsible for the transport of a wide range of compounds, including various drugs, environmental toxins, and endogenous substances such as uric acid [43]. Dysfunction or altered expression of SLC22A8/OAT3 have significant implications for drug disposition, efficacy, and toxicity, as well as for the regulation of endogenous compounds [44]. Lastly, channel proteins form aqueous pores across the membrane, allowing specific ions or molecules to pass through via simple diffusion, driven by electrochemical gradients [21]. SLC26A4 (pendrin) is a protein-coding gene that encodes an anion transporter involved in the regulation of chloride and bicarbonate ions across cell membranes [45]. Mutations in the SLC26A4 gene are associated with Pendred syndrome, an inherited disorder characterized by sensorineural hearing loss, goiter (enlargement of the thyroid gland), and sometimes inner ear abnormalities such as vestibular dysfunction [14,46–49]. Collectively, each of these transport mechanisms plays a crucial role in maintaining cellular homeostasis, regulating nutrient uptake, neurotransmission, ion balance, and many other physiological processes. The diversity of transport mechanisms within the SLC gene family reflects the complexity of cellular transport and the varied requirements of different cell types and physiological conditions.

## 3. SLC Gene Family and Asthma Pathogenesis

Research has shown that certain Solute carrier family (SLC family) genes may be implicated in asthma through several major mechanisms, such as ion transport, inflammation and immune response, oxidative stress, nutrient transport and metabolism, and genetic studies. Of these, ion transport is essential for maintaining the proper function of airway epithelial cells. Dysregulation of ion transport can affect mucus production and airway surface liquid, contributing to asthma symptoms. For example, SLC26A4 as one of the ion transports was reported to be increased in inflammatory lung diseases including asthma, COPD, and various infections [50]. Thus, it is reasonably believed that SLC26A4 inhibition can increase airway surface liquid volume and subsequently prevent inflammatory lung diseases [51]. SLC22A5 has been linked to the transport of carnitine, a molecule involved in fatty acid metabolism and energy production. SLC22A5 was remarkably reduced in patients with severe asthma and has been associated with carnitine and central energy metabolism dysregulation in asthma [52]. SLC family genes also play a role in the transport of antioxidants or molecules related to oxidative stress, which can impact the severity of asthma. Indeed, glucose uptake is required for cell metabolism to provide energy to drive cell proliferation and to support key functions such as the secretion of mucins and surfactants [53]. SLC2A3 (GLUT3) is a glucose transporter that may influence glucose uptake and oxidative stress in the airways [54]. SLC6A2 (NET) and SLC6A3 (DAT) are involved in neurotransmitter transport from the extracellular space into the intracellular compartment, influencing the autonomic regulation of airway tone, likely in asthma [55]. SLC11A1 (NRAMP1) has been shown to be increased in the sputum and serum of patients with asthma, which has been shown to be involved in pathogen resistance, play a role in metal ion transport, and promote anti-microbial pathogen responses [56,57]. Importantly, genetic studies have identified several SLC genes that are associated with asthma susceptibility. For example, genetic mutations in SLC22A5 have been associated with asthma susceptibility and SLC22A5 expression, which can lead to reduced carnitine transport, impacting energy metabolism, and potentially enhancing inflammatory responses in the airways [58,59]. While research on the contributions of genetic mutations in SLC family genes to asthma is limited, it was hypothesized that those mutations could significantly influence the development and severity of asthma through various mechanisms, including altered ion transport, immune regulation, and cellular metabolism. Taken together, understanding the role of SLC family genes in asthma can provide insights into the molecular mechanisms underlying this condition and potentially lead to the development of novel therapeutic strategies targeting specific transporters involved in asthma pathogenesis.

## 4. SLC26A4 Activation and Its Association with Human Diseases

**Figure 3.**
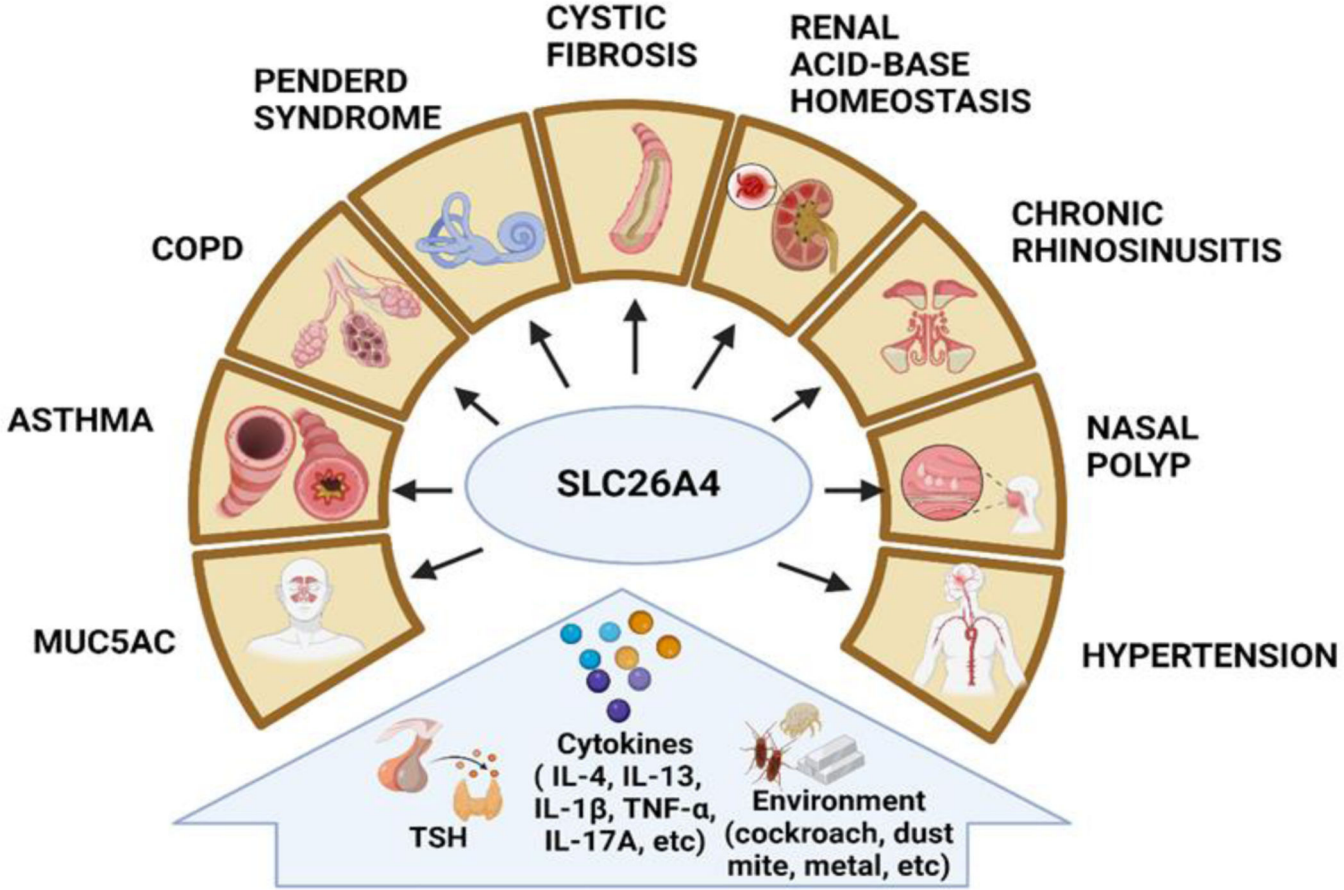
Inducers of SLC26A4 expression and activation and their contributions to diverse diseases.

SLC26A4 is an anion exchanger that mediates bicarbonate (HCO_3_^−^) exchange for chloride (Cl^−^) and is crucial for maintaining pH and salt homeostasis in the kidney, lung, and cochlea [14]. SLC26A4 is a channel protein that forms aqueous pores across cell membranes. SLC26A4 is expressed in various organs and tissues, notably the thyroid gland and inner ear. In the thyroid, SLC26A4 plays a crucial role in transporting iodide ions out of specific cells, which is essential for the synthesis of thyroid hormones [60]. In the inner ear, SLC26A4 helps regulate ion balance, particularly chloride and bicarbonate, which is vital for proper development and function [61]. Its activity is especially significant during inner ear development, potentially influencing the shaping of structures like the cochlea and vestibular aqueduct. More than 150 mutations have been identified in the SLC26A4 gene to be associated with Pendred syndrome characterized by enlargement of the thyroid gland, hearing loss, and other abnormalities of the inner ear, including an enlarged vestibular aqueduct [47]. All of these genetic changes impair or eliminate the activity of SLC26A4, which disrupts ion transport. SLC26A4 is also present in other tissues such as the kidneys, liver, and airway linings, where ongoing research explores its ion transport functions and their implications for physiological processes that are associated with different inducers and diseases (Figure 3).

### 4.1. Induction of SLC26A4 Expression and Activity

In addition to genetic mutations, several other factors also infect SLC26A4 expression and activity, such as thyroid stimulating hormone (TSH), inflammatory cytokines (e.g., IL-4, IL-13, IL-1β, TNF-α, IL-17A), and environmental factors (Figure 3). In the thyroid gland, SLC26A4 expression can be induced by TSH, a hormone released by the pituitary gland that stimulates the production and release of thyroid hormones [60]. TSH acts on thyroid follicular cells to upregulate the expression of SLC26A4, promoting the transport of iodide ions necessary for thyroid hormone synthesis. In turn, inadequate levels of iodine can lead to decreased thyroid hormone synthesis, lower expression of SLC26A4 expression, and impaired iodide transport in the thyroid gland [62]. Inflammatory cytokines such as interleukin-1β (IL-1β) and tumor necrosis factor-alpha (TNF-α) have been shown to induce SLC26A4 expression in various cell types, including airway epithelial cells [63]. These cytokines are produced in response to inflammation and immune activation, and their induction of SLC26A4 may play a role in regulating ion transport and mucin secretion in the airways. For example, IL-13 can induce the expression of SLC26A4 in the apical membrane of bronchial epithelial cells, which may serve as a critical mediator of mucus formation [16]. Similarly, IL-4 can induce SLC26A4 that is responsible for the SCN^−^/Cl^−^ exchange [64]. Thus, both IL-4 or IL-13 can induce the expression of SLC26A4. Furthermore, the epithelial anion transporter SLC26A4 is induced by the combined effects of rhinovirus and IFN-γ during virus infection, regulates airway surface liquid (ASL) thickness, and increases airway reactivity and inflammation in an asthma model [15]. Thus, this study suggests an even broader role for SLC26A4 in the pathophysiology of asthma, especially considering its impact on ASL and its connection with allergic airway illness. A significant finding is that the combination of IL-13 and IL-17A can synergistically enhance SLC26A4 expression in airway epithelial cells [65]. Similarly, the combination of IL-17A and TNF-α can also induce a SLC26A4-mediated complex program that involves pro-found changes in ion transport mechanisms with alteration of airway surface properties, thus perpetuating the proinflammatory airway surface condition [66]. Given that IL-17A is associated with severe asthma and neutrophil infiltration, it suggests that SLC26A4 expression might peak in patients with severe asthma. Furthermore, exposure to environmental pollutants and toxins can influence SLC26A4 expression and activity. For example, exposure to heavy metals such as cadmium, mercury, silica, welding fumes, single-wall carbon nanotubes, and cerium dioxide nanoparticles (CeO_2_NPs) may alter SLC26A4 expression, potentially contributing to tissue dysfunction [67–69]. Additionally, environmental allergens can also contribute to the activation of SLC26A4 [8,70]. For example, repeated intranasal instillation of CeO_2_NPs in the presence of HDM [66] caused the induction of mucin and SLC26A4. Studies from our research group also demonstrated that cockroach allergens can induce SLC26A4 expression in human airway epithelial cells [8]. It is possible that allergens can directly stimulate airway epithelial cells, leading to increased SLC26A4 expression as a part of the immune responses that contribute to airway inflammation and obstruction, and the exacerbation of asthma. Overall, the regulation of SLC26A4 expression and activity is complex and can be influenced by a variety of factors, including hormonal, environmental, and inflammatory stimuli. Further research is needed to fully elucidate the mechanisms underlying SLC26A4 induction and its implications for different diseases.

### 4.2. SLC26A4 Is Linked to Several Human Diseases

Aberrant SLC26A4 expression has been reported in a number of disease models where SLC26A4 is involved in contributing to the patho-mechanisms due to its role as an anion transporter (Figure 3) [47]. While iťs prominently known for its role in the inner ear and thyroid, where mutations in the gene can cause hearing loss and thyroid disorders, respectively, it also plays a significant role in several other diseases. For example, SLC26A4 has been shown to be highly expressed in the nasal mucosa of patients with chronic rhinosinusitis with nasal polyps(CRSwNP) and allergic rhinitis (AR) [65,71,72]. SLC26A4 is also overexpressed in lungs affected by COPD, Bordetella pertussis infection, cystic fibrosis, and rhinovirus infection [15,16,73], and is associated with lipopolysaccharide (LPS)-induced acute lung injury [18]. In addition, SLC26A4 can regulate blood pressure and arterial pH, possibly by participating in the renal regulation of net acid and Cl^−^ excretion [74]. Further studies demonstrated that SLC26A4 mutations protect against the development of high blood pressure through enhanced urinary Na^+^/Cl^−^ excretion, suggesting that SLC26A4 can serve as a potential target for anti-hypertensive drugs [75]. Increased SLC26A4 has also been associated with renal acid-based homeostasis [76]. In the kidneys, SLC26A4 is expressed in specific segments of the renal tubules, including the cortical collecting ducts and the connecting tubules. SLC26A4's activity can influence the excretion of chloride, which indirectly affects acid-base balance. Dysfunction or mutations in the SLC26A4 gene can lead to disturbances in renal acid-base homeostasis, potentially resulting in metabolic acidosis or alkalosis. Most importantly, SLC26A4 has been identified as a common mediator for mucus production in bronchial asthma, highlighting its significance in asthma characterized by mucus hypersecretion and airway obstruction [16].

## 5. SLC26A4 Contributes to the Pathogenesis of Asthma

While the role of SLC26A4 in asthma is not as well understood as in other conditions like hearing loss and thyroid disorders, emerging research suggests its involvement in airway inflammation and mucus production. While SLC26A4 expression is undetectable in normal airway epithelium, its expression is strongly up-regulated in inflammatory airway diseases such as asthma, allergic rhinitis, and COPD [16]. SLC26A4 expression was also significantly upregulated in airway epithelial cells in response to IL-4 and IL-13 [16,64]. Further studies on animal models provided supporting evidence that SLC26A4 is indeed expressed in the lungs of asthma model mice, including those induced by ovalbumin inhalation, IL-13 inhalation, and IL-13 transgenic mice [16,64,77]. Specifically, SLC26A4 is primarily expressed in the apical side of airway epithelial cells in these mice and non-ciliated airway epithelial cells seem to be the main cell type expressing SLC26A4 in response to IL-4/IL-13. Additionally, the expression of SLC26A4 appears to be enhanced in both acute and chronic asthma models, indicating its potential involvement in the pathophysiology of asthma [77]. Consistently, SLC26A4-deficient mice showed less allergen-induced airway hyper-reactivity and inflammation in relative to control mice [15], and reduced lung inflammation in response to Bordetella pertussis [17]. SLC26A4 expression was significantly increased by LPS stimulation by both in vitro and in vivo analyses, and inhibition of SLC26A4 by the small molecule YS-01 dramatically attenuated LPS-induced lung injury [19]. Additionally, SLC26A4 regulates the pH of the airway surface liquid, which is crucial for optimal ciliary function and pathogen defense. Overall, while the precise role of SLC26A4 in asthma requires further investigation, accumulating evidence suggests its involvement in the pathophysiology of inflammatory airway disease.

**Figure 4.**
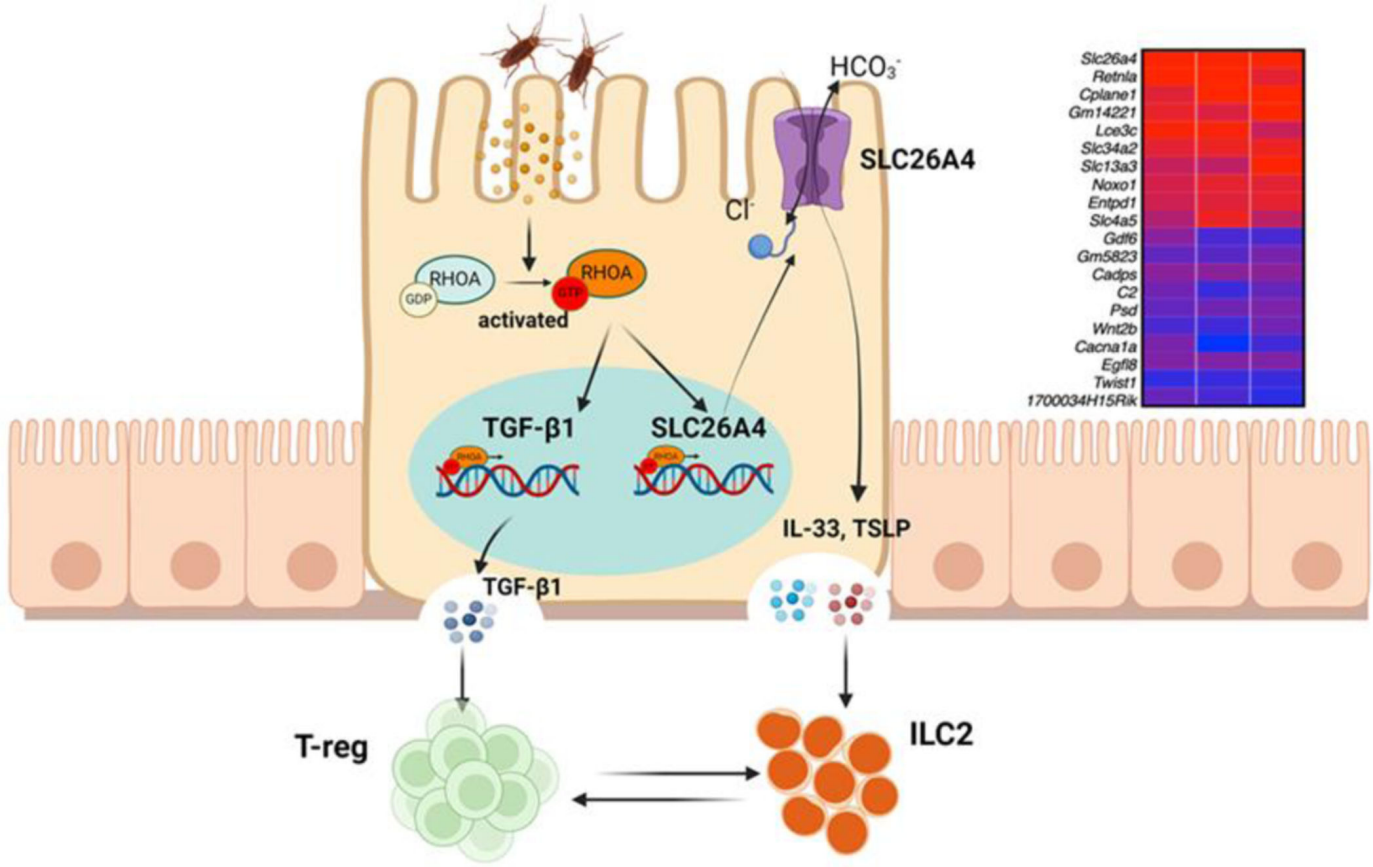
Axis of RhoA-SLC26A4 protects against airway inflammation through the immuno-imbalance between Tregs and ILC2s. Up-right: Top 10 up or down-regulated genes by RhoA in AT2 cells. Transforming Growth Factor-beta 1 (Tgf-β1).

**Figure 5.**
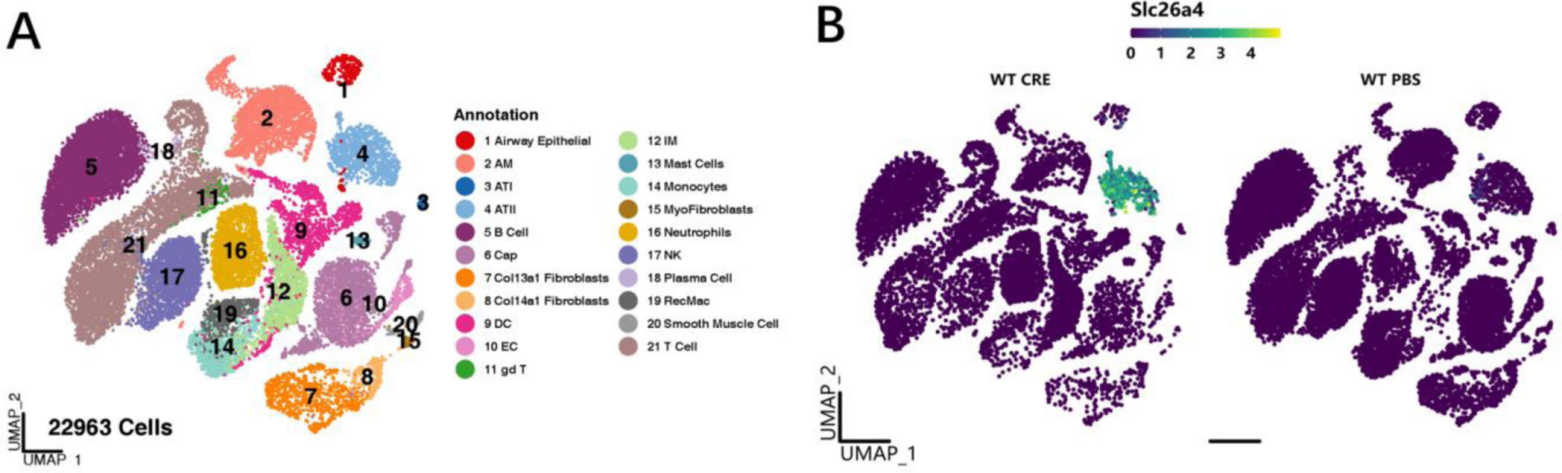
Single cell RNA-Seq analyses of lung tissues from asthma mouse model and controls. (**A**) A total of 22963 cells from 16 mice (8 for asthma and 8 for control) were finally annotated to 21 kinds of cells including epithelial, mesenchymal, and immune cells. (**B**) SLC26A4 was highly and uniquely expressed in AT2 cells after cockroach allergen treatment. Log2FC = 6.71 with adj-*p* value < 0.0001, based on pseudobulk and edgeR pipeline with likelihood ratio test.

Our recent RNA-seq analyses on AT2 cells identified SLC26A4 as the most up-regulated gene in Ras homolog family member A (RhoA)-deficient AT2 cells (Figure 4) [8]. The small GTPase RhoA and its downstream effectors are critical regulators in the pathophysiological processes of asthma and a promising therapeutic target for the treatment of asthma [78]. SCL26A4 has been shown to be increased in endobronchial biopsies from patients with asthma [64,79,80]. We reported for the first time that there was a significant increase in the expression of SLC26A4 in AT2 cells of asthmatic patients compared with healthy controls. Importantly, compared with healthy controls, mild/severe asthmatics had higher levels of serum SLC26A4, which were correlated with pulmonary function parameters. Further in vitro analysis indicated that SLC26A4 was significantly up-regulated in the primary bronchial epithelial cells after treatment with cockroach allergen. While the exact role of SLC26A4 regarding the pathogenesis of asthma is still unclear, our study suggests a novel mechanism that the RhoA-SLC26A4 axis in AT2 cells protects against allergic airway inflammation through the immuno-imbalance between augmented Treg and reduced ILC2 cells (Figure 4). Very recently, we performed single cell RNA-Seq (scRNA-Seq) analyses of lung tissues from asthma mouse models and controls. A total of 22,963 cells from 16 mice (8 for asthma and 8 for control) were finally annotated to 21 kinds of cells including epithelial, mesenchymal, and immune cells (Figure 5A). SLC26A4 was highly and uniquely expressed in AT2 cells after cockroach allergen treatment (Figure 5B, log_2_FC = 6.71 with adj-*p* value < 0.0001, based on pseudobulk and edgeR pipeline with likelihood ratio test) [81,82], highlighting a possible role for SLC26A4 in AT2 cells in allergic airway inflammation.

**Figure 6.**
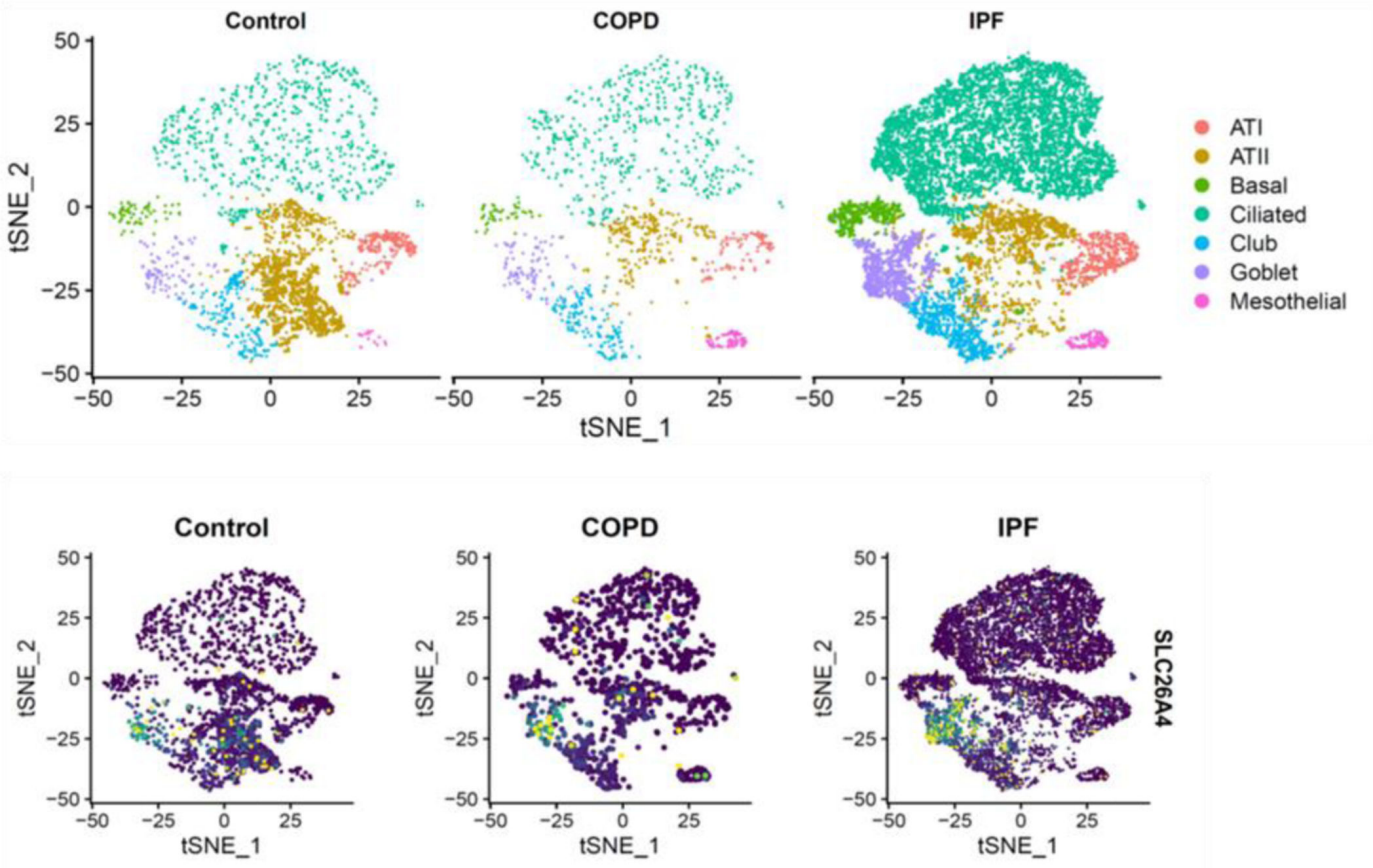
Single cell RNA-Seq analyses on public datasets from the Gene Expression Omnibus (GEO) database GSE136831 generated with whole lung dissociates from idiopathic pulmonary fibrosis (IPF), COPD and control lungs.

Given that SLC26A4 is involved in the transport of chloride, iodide, and bicarbonate in various tissues, it is very likely that SLC26A4 in AT2 cells may play a role in ion transport and regulation of fluid balance, pH regulation, and modulation of surfactant secretion. Thus, further study on the precise role of SLC26A4 in AT2 cells is essential and holds promise for understanding the detailed mechanisms of lung biology and potential therapeutic targets. In addition, we found that SLC26A4 is also highly expressed in goblet cells of patients with COPD and idiopathic pulmonary fibrosis (IPF) as compared to control lungs when analyses were performed on public datasets from the Gene Expression Omnibus (GEO) database GSE136831 with scRNA-Seq of whole lung dissociates from IPF, COPD, and control lungs (Figure 6) [83].

Intriguingly, SLC26A4 has been previously identified as a common mediator for mucus production in bronchial asthma [16]. Collectively, the upregulation of SLC26A4 in specific cell types, such as airway epithelial cells and goblet cells, is driven by inflammatory cytokines, allergens, and oxidative stress, involving multiple signaling pathways. It would be of interest to explore the precise role of SLC26A4 in goblet cells and AT2 cells and investigate their impact on mucus production and pathology in asthma.

## 6. SLC26A4, A Potential Therapeutic Target for Asthma

SLC26A4 has been identified as a potential alternative therapeutic target for asthma exacerbations, and inhibition of SLC26A4 has been considered a validated approach to increase airway surface liquid volume, and attenuate airway hyperresponsiveness in asthma [8,48,73]. Several inhibitors of SLC26A4 have been developed and found to be effective in suppressing airway hyperresponsiveness, airway inflammation, and some asthma-related phenotypes. For example, high-throughput screening identified tetrahydropyrazolopyridine and pyrazolothiopenesulfonamide as potential SLC26A4 inhibitors by binding to SLC26A4 and interfering with its ion transport activity. These inhibitors have been shown to reversibly inhibit SLC26A4-facilitated Cl^−^ exchange with SCN^−^, I^−^, NO_3_^−^, and HCO_3_^−^, and increase ASL depth caused by IL-13 treatment [73]. The small compound PDSinh-A01 has been shown to inhibit Cl^−^/HCO_3_^−^ exchange and increase ASL thickness in IL-13-treated primary human bronchial epithelial cell cultures [73]. YS-01 (2-(4-(tert-butyl)phenyl)-4-(thiophen-2-ylmethylene)oxazol-5(4H)-one), a novel SLC26A4 inhibitor identified by the high-throughput screening of 54,400 synthetic compounds, showed strong therapeutic effects on allergic inflammation in a mouse model of OVA-induced asthma [84]. Mechanistically, YS-01 was found to reduce airway hyperresponsiveness and airway inflammation via the inhibition of the SCN2/Nuclear Factor-kappa B (NF-kB) pathway [84]. Furthermore, YS-01 showed protective effects against IL-4– and IL-13–induced goblet cell hyperplasia and ASL depletion. Of interest, AS-01 showed more inhibition of SLC26A4 activity than PDSinh-A01. Furthermore, YS-01 inhibited both human and mouse SLC26A4-mediated Cl^−^/I^−^ exchange with similar potency. Additionally, we used NPPB (5-Nitro-2-(3-phenylpropylamino)benzoic acid), an inhibitor of anion channels that has been shown to suppress the SLC26A4-induced CI^−^ uptake [85] and SLC26A4 activity [86], and found that pretreatment of mice with NPPB abrogated cockroach allergen-induced airway inflammation and Th2-associated cytokines. Taken together, these findings indicate that SLC26A4 may be a useful target for the treatment of allergic asthma. However, the direct implications of NPPB on asthma symptoms and related mechanisms remain poorly understood. A very recent study has explored the structures of SLC26A4 by applying cryo-electron microscopy and identified two anion binding sites, and functional analyses suggest that two anion binding sites are involved in anion exchange [14]. These results reveal directions for understanding the mechanisms of anion selectivity and exchange, and also establish a foundation for the development of small-molecule inhibition of SLC26A4.

## 7. Conclusions

In this review, we explore the diverse roles of the SLC gene superfamily in cellular transport mechanisms, focusing on the facilitative, active, and secondary active transporters, including antiporters, symporters, co-transporters, and channel proteins. Among these, the channel protein SLC26A4 emerges as a pivotal player in ion transport and homeostasis, underscoring its importance within the SLC gene family. We detail the triggers that can induce SLC26A4 expression and activity, such as genetic mutations, TSH, and inflammatory cytokines like IL-4, IL-13, IL-1β, TNF-α, and IL-17A, alongside environmental factors. Furthermore, we explore how activated SLC26A4 is associated with various human diseases, including CRSwNP, COPD, asthma, cystic fibrosis, lung injury, hypertension, and renal acid-base homeostasis. SLC26A4 has been identified as a key mediator in regulating airway surface liquid thickness, mucus production, airway hyperresponsiveness, and inflammation in asthma. This positions SLC26A4 as a promising therapeutic target for asthma, where inhibition has been shown to enhance airway surface liquid volume and reduce airway hyperresponsiveness. Nevertheless, a more comprehensive exploration within the SLC26 family context is essential to fully understanding its critical role in ion transport and homeostasis. Continued research is imperative to unravel SLC26A4’s intricate involvement and potential as a therapeutic target, especially considering its significant connections to respiratory disorders such as asthma.
